# Nutritional Strategies to Offset Disuse-Induced Skeletal Muscle Atrophy and Anabolic Resistance in Older Adults: From Whole-Foods to Isolated Ingredients

**DOI:** 10.3390/nu12051533

**Published:** 2020-05-25

**Authors:** Ryan N. Marshall, Benoit Smeuninx, Paul T. Morgan, Leigh Breen

**Affiliations:** 1 School of Sport, Exercise and Rehabilitation Sciences, University of Birmingham, Edgbaston, Birmingham B15 2TT, UK; RNM857@bham.ac.uk (R.N.M.); benoit.smeuninx@monash.edu (B.S.); p.t.morgan@bham.ac.uk (P.T.M.); 2 Medical Research Council-Versus Arthritis Centre for Musculoskeletal Ageing, University of Birmingham, Edgbaston, Birmingham B15 2TT, UK; 3 Monash Institute of Pharmaceutical Sciences, Monash University, Parkville, VIC 3052, Australia

**Keywords:** skeletal muscle, atrophy, nutrition, disuse, ageing, inactivity, metabolism, protein

## Abstract

Preserving skeletal muscle mass and functional capacity is essential for healthy ageing. Transient periods of disuse and/or inactivity in combination with sub-optimal dietary intake have been shown to accelerate the age-related loss of muscle mass and strength, predisposing to disability and metabolic disease. Mechanisms underlying disuse and/or inactivity-related muscle deterioration in the older adults, whilst multifaceted, ultimately manifest in an imbalance between rates of muscle protein synthesis and breakdown, resulting in net muscle loss. To date, the most potent intervention to mitigate disuse-induced muscle deterioration is mechanical loading in the form of resistance exercise. However, the feasibility of older individuals performing resistance exercise during disuse and inactivity has been questioned, particularly as illness and injury may affect adherence and safety, as well as accessibility to appropriate equipment and physical therapists. Therefore, optimising nutritional intake during disuse events, through the introduction of protein-rich whole-foods, isolated proteins and nutrient compounds with purported pro-anabolic and anti-catabolic properties could offset impairments in muscle protein turnover and, ultimately, the degree of muscle atrophy and recovery upon re-ambulation. The current review therefore aims to provide an overview of nutritional countermeasures to disuse atrophy and anabolic resistance in older individuals.

## 1. Introduction

Human skeletal muscle mass and strength are of great importance for maintaining cardio-metabolic health and locomotion in older age [1]. With advancing age, a loss of muscle mass and strength is observed (sarcopenia), increasing the risk of falls, fractures and mortality [2]. The age-related decline in muscle mass and strength occurs at an annual rate of ~1% and 3%, respectively, from the fifth decade of life onwards [3,4]. Sarcopenia is estimated to affect as many as ~32 million older individuals across Europe in the coming decades [5], at an annual cost to the UK health services of ~£2.5 billion (1.2% of GDP). Therefore, developing strategies to counteract this phenomenon are essential to extend the number of years lived in good health, as well as reduce the burden on health care systems and the global economy.

**Figure 1:**
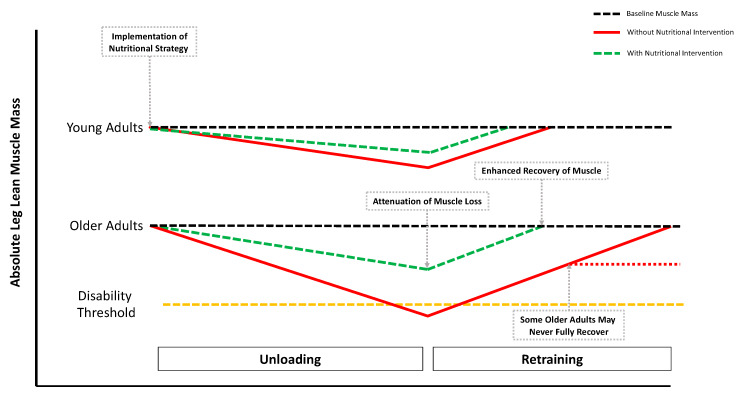
Skeletal muscle atrophy younger and older adults in response to a period of unloading and a subsequent period of exercise rehabilitation. The black dotted lines indicate phenotypical differences in absolute levels of muscle mass. The yellow dotted line indicates a level in which low levels of leg lean mass results in disability and reduced functional ability (i.e., disability threshold). The red line indicates traditional trajectory in loss of muscle mass observed by disuse. The green dotted line indicates the potential of a nutritional strategy to offset muscle loss during disuse and improve rates of recovery following reambulation and rehabilitation. Importantly, the red dotted line indicates a proportion of older adults may not return to pre-disuse levels of muscle mass, particularly in the absence of aggressive retraining (Adapted from Perkin et al. 2016 [17]).

The maintenance of human skeletal muscle hinges on the dynamic balance between muscle protein synthesis (MPS) and muscle protein breakdown (MPB). To increase skeletal muscle mass, MPS must exceed MPB, which can be achieved by performing regular resistance exercise training (RET) in combination with adequate protein nutrition [6,7]. However, whilst young individuals demonstrate a pronounced response to these anabolic stimuli, a blunted response has been observed in older adults [8]. Indeed, maximal stimulation of MPS occurs at much lower relative dosages in younger adults (~0.24 g·kg^−1^·BW), compared with older adults (~0.4 g·kg^−1^·BW) [9]. The phenomenon of “anabolic resistance” in older adults was first established by Volpi and colleagues, who infused older adults intravenously with an amino acid-glucose mixture thereby bypassing splanchnic uptake [10]. Following a ~3-h infusion period, a blunted MPS response was observed in older compared with younger adults [10]. This was subsequently confirmed in seminal work by Cuthbertson et al. [11] following an oral dose–response (i.e., 0, 2.5, 5, 10, 20 and 40 g) of crystalline essential amino acids (EAAs). The authors observed a maximally stimulating effect of ~10 g of EAAs in younger adults; however, a large (~40 g) bolus failed to elicit similar increases in MPS to the smaller ~10 g provision observed in younger adults and is likely explained by a decrease in postprandial anabolic sensitivity and diminished mTORC1 phosphorylation in older adults [11]. This age-related anabolic resistance is likely an important driving factor in age-related muscle loss and, importantly, is exacerbated during intermittent periods of musculoskeletal disuse and reduced physical activity (PA) [12,13]. Furthermore, anabolic resistance can also occur in younger adults following brief periods of disuse [14,15], although evidence suggests that younger individuals recover losses in muscle mass and strength at a quicker rate than older individuals upon re-ambulation (Figure 1) [16,17]. These transient and intermittent episodes of inactivity result in significant muscle wasting, predominantly in the lower limbs, affecting activities of locomotion and daily living. Indeed, it has been shown that five days of bed-rest (BR) results in quadriceps muscle loss equivalent to ~5 years of ageing [18]. This disuse-induced accelerated model of sarcopenia leads to a downward spiral of health, resulting in a greater risk of hospital (re)admission and an increased length of hospital stay [19,20]. In 2017, ~18.7 million adult hospital admissions were recorded, of which ~7.6 million were aged 65 and over [21]. Simultaneously bed occupancy rates soared to nearly 90% in the UK [22]. As a result, minimising muscle deterioration during disuse events in older individuals will likely reduce the length of hospital stay, complications following discharge and readmission rates to inpatient facilities.

## 2. Experimental Models of Disuse and Inactivity

Several experimental models have been used to explore the effects of reduced PA and/or disuse on human skeletal muscle mass. The use of step-reduction (SR) in experimental research is highly relevant to numerous situations of old age in which activity levels are reduced, including transient periods of inactivity (e.g., illness and elective surgery) [23]. Previous investigations using SR typically reduce daily step-count to ≤2000/day over 7–14 days [12,15,24]. To place this in context, ~750 steps/day represents typical ambulatory activity during an acute hospital stay [12,15,23]. SR studies consistently show a reduction in postprandial [12] and free-living MPS [15,24], as well as marked reductions in muscle mass [12]. Another model of disuse atrophy, with clinical relevance, is unilateral limb immobilisation (ULI), achieved by the casting (or bracing) of a given limb over days to weeks [14,25], with the non-immobilised contralateral limb often serving as a within-subject control [26]. During ULI, limbs can be immobilised at specific angles to investigate the effects of passive shortening or lengthening on muscle fibres, thereby inducing significant muscle atrophy and strength loss. Mechanistically, ULI has consistently been reported to impair postabsorptive [27], postprandial [14,28] and free-living MPS [29] and elevate markers of proteolysis [30,31]. The use of bed-rest (BR) in experimental research has also provided an excellent platform to enhance understanding of the impacts of reduce PA on skeletal muscle health, particularly in older individuals [32,33,34]. This mode of disuse is of particular importance as older individuals experience a greater number of hospital admissions that typically last for 5–6 days on average and are particularly vulnerable to metabolic complications. Experimental studies of BR typically occur over 5–14 days [32,33,35], but ~28 days [36,37] and ~90 days of BR with a 6° head-down tilt (to mimic spaceflight) [38] have also been used. Similar to other disuse models, BR has been reported to impair postabsorptive [39], postprandial [40] and free-living MPS [41] and elevate markers of proteolysis [18], as well as reduce muscle mass, strength and function [42].

**Figure 2:**
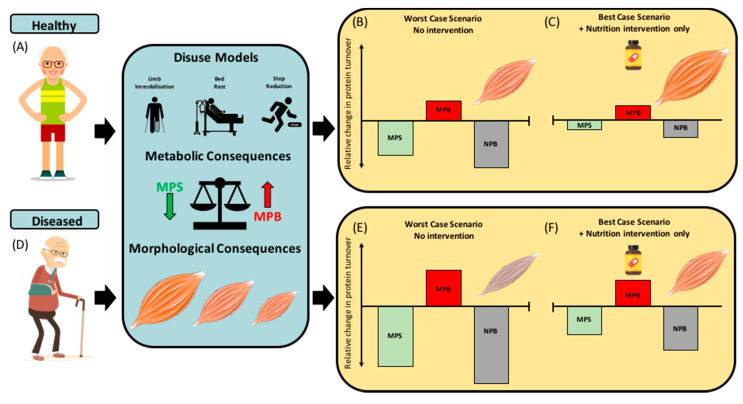
Illustration of the effects of disuse events on mechanisms of disuse atrophy in older healthy and diseased phenotypes and theoretical potential for nutritional interventions to mitigate these detrimental effects. (**A**–**C**) The effects of disuse (bed-rest and unilateral limb immobilisation) and inactivity (step reduction) on muscle protein synthesis (MPS), breakdown (MPB), net protein balance (NPB) and muscle mass in healthy older adults, with the worst-case scenario (i.e., disuse with no nutritional strategy) and best-case scenario (i.e., with potentially effective nutritional strategy). (**D**–**F**) The effects of disuse and inactivity on MPS, MPB, NPB and muscle mass in older adults with compromised health status (frailty, malnutrition syndrome, cachexia and chronic inflammatory disease) and the worst- and best-case scenario. With nutritional intervention-only, the best-case scenario may result in more positive NPB (albeit still negative) and an attenuated rate of muscle atrophy. NB: The improvement in NPB outlined in the best-case scenario panels is driven primarily through nutritional stimulation of MPS, whereas nutrient-induced MPB suppression plays a lesser role (due to the absence of clear evidence demonstrating MPB suppression with nutritional interventions during disuse).

Undoubtedly, studies implementing these experimental models of disuse and inactivity have furthered our understanding of the mechanisms regulating muscle deconditioning during unloading. However, questions remain around the clinical relevance and translation of these studies to patient populations. Specifically, whilst younger and older research volunteers are likely to be in good health, older patients admitted to hospital often suffer from illness and trauma/injury in the presence of several other comorbidities. This could, therefore, render many older hospital patients in a state of hyper-metabolism or auto-cannibalism, resulting in excessive loss of muscle mass that may not be apparent in pre-clinical studies in healthy volunteers [43,44,45]. RET and protein nutrition are undoubtedly the most potent non-pharmacological interventions to maintain, or even increase, muscle mass and function with advancing age [46]. Nevertheless, as performing RET may not be viable during acute episodes of disuse, such as SR, ULI and BR, the purpose of this review is to summarise the literature on nutritional strategies to minimise the loss of skeletal muscle mass and function in humans during different experimental models of disuse. As such, nutritional interventions with the potential to offset disuse atrophy may be a preferred approach in some clinical settings. Given the knowledge that alterations in MPS appear to be a primary driver of disuse atrophy in young and old (at least in healthy individuals), nutritional stimulation of MPS may be an effective way to arrest the decline in muscle mass. Due to difficulties in the direct measurement of in vivo MPB rates, little is known about the temporal time-course of MPB during different models of disuse and the overall contributing role of MPB in disuse atrophy. As such, the effect of nutritional interventions on disuse-induced MPB are relatively unknown. Hence, the primary focus of nutritional interventions to attenuate disuse atrophy in older adults is on stimulation of MPS (i.e., overcoming anabolic resistance), the success of which would likely differ between healthy older adults and those with a compromised health status (Figure 2).

## 3. Nutritional Strategies during Disuse

### 3.1. Whole Food and Mixed-Meal Approaches

The “food-first” (or “whole-food”) approach, unlike isolated protein components (e.g., whey protein and casein protein, discussed below), contain a variety of other non-protein derived nutrients that may facilitate intramuscular anabolic signalling, MPS and tissue remodelling [47]. This approach has gained interest in human metabolic research where, compared with studies using isolated proteins, several recent investigations have focused on the muscle anabolic response following consumption of protein-rich whole-foods, such as beef [48,49,50], eggs [51,52], skimmed milk [50], poultry [53,54] and other mixed macronutrient meals [55,56,57,58]. However, an in-depth discussion of the food-first approach vs. isolated proteins is beyond the scope of this review and has been discussed elsewhere [47,59].

It has recently been suggested that a high-quality protein whole-food approach can support muscle mass throughout the lifespan [59], likely due to nutrient density, and non-nutritive components that may facilitate muscle remodelling compared to pure, isolated protein sources [51]. High protein quality, as measured by the protein digestibility-corrected amino acid score (PDCAAS) [60], the digestible indispensable amino acid score (DIAAS) [61] or the leucine amino acid reference ratio (Leu-AARR) [62], are critical in the promotion of MPS and long-term accrual of muscle mass [59]. However, as a blunting of the MPS response to protein provision is observed with advancing age [56,63,64,65], one might question whether providing protein-dense whole-foods is desirable in older or clinical populations considering their prolonged satiating effect and relatively slow rate of gastric emptying [66,67], which may negatively affect overall daily dietary protein intake in a population already prone to protein malnourishment. Indeed, it has previously been shown that older adults in an acute care facility only consumed ~10 g of a commendably large ~40 g serving of protein at each meal [68]. The authors postulated that food taste, temperature, texture and preferences all play a role in protein consumption within acute medical inpatient facilities. Furthermore, similar issues with sub-optimal dietary protein consumption have also been observed in older adults undergoing elective hip/knee surgery [69], whereby only ~30% of meal-time protein was consumed [9], clearly identifying the need to consider other strategies to ensure sufficient protein to attenuate muscle loss is consumed by older adults undergoing protracted disuse events. More specifically, following short-term hospitalisation in patients with elective hip/knee surgery, it was shown that, despite patients being provided with ~0.8 g·kg^−1^·BW·day^−1^ of protein in self-selected hospital meals, actual protein consumption fell below 0.6 g·kg^−1^·BW·day^−1^. This was accompanied by a ~4% reduction in thigh cross-sectional area [69,70]. Furthermore, a recent seven-day BR protocol, with a diet composed of traditional inpatient mixed macronutrient meals (~1.0 g·kg^−1^·BW·day^−1^ [~70% animal, ~30% plant proteins]) failed to attenuate the decline in leg lean mass and strength in healthy older adults [71]. Although total provided protein intake in both investigations provided the current RDA (0.8 g·kg^−1^·BW·day^−1^), they fell short of the current clinical recommendations for older individuals during hospitalisation (~1.6 g·kg^−1^·BW·day^−1^) [72]. Therefore, the ingestion of additional volume of mixed meal proteins potentially may have offset muscle loss but may also come at the expense of increased and prolonged satiety. Furthermore, it is also pertinent to note that the amount of protein per kcal of food is less. Therefore, besides satiety, the subdivision of macronutrients from the total daily caloric intake is likely sub-optimal.

An often-overlooked aspect of protein-rich whole-food approaches, when compared with isolated sources, is the texture of the foods. This is particularly important for older adults who may have impaired food chewing capabilities [73]. Evidence suggests that the partial processing via mincing of protein-rich lean meats, such as beef, has been shown to increase protein digestion and absorption in older adults, resulting in greater postprandial protein incorporation when compared with ingestion of whole beefsteak [49]. However, it is important to note that, in this study, MPS rates did not differ between these conditions when assessed over a 6-h postprandial period [49]. Nevertheless, this method of protein processing and/or enhanced mastication of ingested foods may be a novel method of increasing the amount, digestibility, and absorption of amino acids (AA) from protein-rich whole foods, which may be especially important for older adults, particularly those undergoing disuse or inactivity. Indeed, novel data in elderly volunteers who were edentulous, found a significantly reduced postprandial rise in circulating plasma EAAs following a serving of lean beef meat, which subsequently resulted in an ~18% reduction in whole-body protein synthesis compared with older adults who had healthy natural dentition [74]. This poses the question as to whether older adults may need softer textured or partially processed foods to increase protein consumption and, therefore, postprandial AA availability and muscle anabolism. A comparison of distinct protein whole-foods with divergent AA delivery profiles in healthy younger adults was conducted by Burd et al. in response to protein dose-matched, skimmed milk or lean beef [50]. Although beef consumption resulted in a ~7% greater AA availability over the initial 2 h postprandial period, skimmed milk consumption stimulated a greater MPS response during this time frame. However, there were no differences in MPS stimulation between beef and milk ingestion when assessed over a 5 h postprandial period [50]. Nevertheless, the trade-off between ease of ingestion and postprandial AA availability between these common protein-rich food sources could present different muscle anabolic responses in older adults during periods of disuse.

Protein-rich whole-foods undoubtedly promote muscle anabolism in the acute postprandial period (~2–6 h) in healthy younger [51,52], older [49,50,75] and inflammatory disease patients [56]. Therefore, the role of high-quality whole-food protein mixed meals in maintaining muscle mass should provide a solid nutritional foundation in which supplementation can support (Figure 2). The anabolic concept of the food matrix is principally demonstrated in the form of whole-egg and isonitrogenous amounts of egg-whites consumed post-RET [51]. Van Vliet and colleagues observed a greater post-RET stimulation of MPS following whole-egg consumption, than that of egg whites [51]. Interestingly, the authors detected no differences in postprandial leucine availability or intracellular anabolic signalling [51]. The authors therefore speculated that whole eggs are more than the sum of their parts. In particular, non-nutritive components [i.e., bioactive peptides, microRNAs, lipids (phosphatidic acid, palmitic and DHA) and minerals] may orchestrate the greater MPS response observed with whole-eggs and not egg white consumption [51]. Follow-up investigations from the same group have established whole-egg consumption results in greater co-localisation of mTOR-Rheb complexes to the lysosome following RET than that of egg whites [52]. Nevertheless, the anabolic role of the food matrix is yet to be established in older adults. Accordingly, older adults should seek to consume the recommended dietary protein intake (~1.6 g·kg^−1^·BW·day^−1^), rich in nutrient-dense foods to attenuate loss of muscle mass during disuse events. Unfortunately, in many disuse scenarios, the practicality of large daily whole-food consumption in older adults is low. Therefore, the addition of rapidly digested isolated proteins may be necessary to enhance protein quality, quantity and the net muscle anabolic effect of typical sub-optimal protein-containing meals in older and clinical populations.

### 3.2. Supplemental Protein Sources

Whey protein (WP) and casein protein (CP) nutritional supplements are produced by separating proteins from their naturally occurring whole-food derivative (i.e., milk). Studies investigating the metabolic profiles of dairy-derived proteins demonstrate the rapid digestion and absorption kinetics of isolated WP compared with the more slowly digested casein [76,77,78] and complete whole-milk proteins [79,80]. Indeed, evidence exists to suggest WP possesses superior muscle anabolic properties compared with casein. Specifically, following consumption of WP, the increase in MPS is greater than casein, potentially due to the superior AA profile and postprandial leucinemia of WP [77]. However, not all authors have observed a benefit of WP over its whole-milk component for muscle anabolism, despite temporal differences in aminoacidemia [81,82]. This may be partly due to the whole-food matrix and added macro-micronutrient value of whole milk, which may facilitate the potent and rapid increase in MPS. Moreover, the measurement duration, protein dosage and synergistic effect of RET likely influences the subtle differences or similarities observed between whole milk protein, isolated WP, and CP derivatives on acute measures of postprandial MPS. Nevertheless, high-quality leucine-rich WP supplementation has been shown to induce a greater postprandial MPS response in both younger and older adults compared with other dose-matched isolated protein sources (e.g., soy, casein and wheat) [78,83] under conditions of rest and exercise [78]. Evidence also exists to support the use of WP during inactivity and disuse. Indeed, recent investigations have found WP to be effective in offsetting BR-induced muscle deterioration in older adults [71]. Furthermore, Arentson-Lantz et al. (2019) demonstrated that replacing whole-food mixed meal proteins with WP (i.e., improving protein *quality*), attenuated the decline in whole-body lean tissue during seven days of BR. Importantly, dietary protein intake was relatively low in this study (~0.9 g·kg^−1^·BW·day^−1^) and, therefore, more representative of the eating habits of older adults within inpatient facilities [69].

Others have attempted to navigate the issue of dietary protein malnutrition during BR with the implementation of blended mixed protein complete meal replacements [WP, CP, pea protein and soy protein] [41]. Specifically, Dirks et al. (2019) provided volunteers with a standardised diet of ~1.2 g·kg^−1^·BW·day^−1^ of dietary protein, introduced via nasogastric tube by four intermittent protein boluses or via a 24-h continuous feeding approach [41]. Interestingly, the findings suggested that the delivery method of dietary protein did not influence the negative consequences of BR on skeletal muscle mass, strength and metabolic function. In addition, it is of interest to note that the total amount of dietary protein (~1.2 g·kg^−1^·BW·day^−1^) was insufficient at protecting losses in muscle mass [41].

During ULI, the use of isolated dietary proteins have been reported to have no effect on skeletal muscle mass and strength loss in healthy older males when compared with a placebo [84]. Specifically, Dirks et al. (2014) used a five-day whole-leg cast immobilisation approach with and without WP supplementation (2 × ~21 g daily [10.6 g EAAs, 2.8 g leucine per drink]), which increased daily protein intake from ~1.1 to ~1.6 g·kg^−1^·BW·day^−1^. Others have also shown no beneficial effect of protein supplementation during more extended periods of ULI (~14 days), in healthy middle-aged males [85], albeit with lower daily supplemental strategies (1 × 20 g). Mitchell et al. (2018) observed no benefit of a single daily bolus of ~20 g milk protein with breakfast on preventing loss of muscle mass and strength during a habitually skewed dietary protein intake of 1.0 g·kg^−1^·BW·day^−1^ (breakfast 25%, lunch, 25% and dinner 50% of energy and protein requirements) to represent typical eating habits of older individuals [85]. Although current data are inconclusive on the effects of WP during ULI, further investigation into other WP strategies (e.g., dosage (~40 g) [86], timing [87], pre-sleep consumption [88], pattern distribution [89] and additional free-form leucine [90]) could offer protection against disuse atrophy.

To date, there has only currently been one randomised controlled trial (RCT) of the influence of dietary protein during an SR model [24]. Oikawa et al. (2018) found that a high quality, high-protein diet (~1.6 g·kg^−1^**·**BW·day^−1^) during SR (~750 steps day^−1^) and a ~500 Kcal energy deficit for two weeks, failed to attenuate muscle atrophy in older adults. Specifically, the provision of two daily WP drinks (providing ~30 g protein each (15.4 g EAA, 4.3 g leucine) or a protein-matched hydrolysed collagen peptide drink (4.5 g EAA, 0.8 g leucine) during SR failed to attenuate the reduction (~14%) in free-living MPS [24]. An important observation from this study is that a seven-day transition from energy balance to energy restriction evoked a decline in MPS (~12%), even in the presence of double the current protein RDA (~1.6 g·kg^−1^**·**BW·day^−1^). Alarmingly, Oikawa et al. (2018) also found that, following a seven-day period of re-ambulation, MPS was not fully restored to levels pre-intervention levels, highlighting the importance for an appropriately prescribed rehabilitation and nutrition program following a period of disuse in older adults [24]. Nonetheless, whilst WP supplementation failed to prevent the loss of lean tissue during SR, WP did promote a greater recovery of lean tissue upon reambulation [24].

Although isolated proteins have been shown acutely to stimulate postprandial MPS and augment the muscle anabolic response to RET, these isolated ingredients, in particular high-quality WP, may be relatively ineffective at attenuating muscle atrophy during disuse in older adults. This may be due to the relatively good health of older volunteers in these studies, or possibly the relatively high level of total combined daily dietary protein intake (meals, plus supplementation) provided in these studies (~1.6 g·kg^−1^**·**BW·day^−1^ [24,84]). Therefore, greater relative daily amounts (>2 g·kg^−1^**·**BW·day^−1^), combined with novel isolated intact protein blends as well as protein sources containing high relative proportions of di- and tripeptides (i.e., protein hydrolysates), may be needed to facilitate greater protective effects on muscle during disuse. Indeed, whilst evidence is limited, protein hydrolysates have been observed to be superior to intact proteins, largely due to decreased splanchnic extraction, increased postprandial AA bioavailability and rapid appearance of EAAs, in particular leucine, into circulation [78,91,92,93]. Moreover, WP hydrolysates have also been observed to augment a greater acute postprandial MPS response compared to intact protein sources in younger and older adults [78,91,93]. However, ultimately, more chronic investigations are required to assess the longer-term anabolic potential of protein hydrolysates compared with intact (whole) proteins, particularly during periods of disuse and inactivity. Nevertheless, protein supplementation may, however, be of use to older adults with compromised health status (i.e., pre-existing anabolic resistance) or those who consume low levels of dietary protein (i.e., <0.8 g·kg^−1^**·**BW·day^−1^) [94], which is particularly prevalent during illness/hospitalisation.

### 3.3. Crystalline Amino Acids

During periods of inactivity and disuse, the consumption of whole-food mixed meals or even isolated protein sources may not be feasible, primarily due to the aforementioned issues of appetite and satiety regulation that are common in older adults. Therefore, constituent free-form AAs as either a complete EAA profile (i.e., all nine EAAs) or individual AAs (i.e., leucine) that possess anabolic and anti-catabolic properties may be an effective and energetically efficient in offsetting muscle atrophy. The following section discusses the efficacy of AA supplementation in a range of modes of disuse and inactivity.

#### 3.3.1. Essential Amino Acids

Isocaloric consumption (15 g) of EAAs (2.79 g leucine) in older individuals has been shown to result in a >30% increase in MPS compared to a protein-matched ~15 g WP (1.75 g leucine) [95], likely due to the higher proportion of EAAs and more rapid digestion and absorption of crystalline EAAs. A follow-up investigation found that, when matched for EAA content, ~15 g of WP resulted in a greater accrual of muscle proteins compared to 6.7 g of EAA, overall indicating that the content of EAA within WP is likely not the sole explanation for its anabolic superiority to pure EAAs [96]. As discussed above, an important consideration regarding protein nutritional strategies in older adults is the dosage required for maximal MPS stimulation (EAA, ~15 g, WP, >30 g), and their respective effects on appetite and satiety [97]. Recent evidence comparing an EAA-matched dose of WP ~15 g (7.5 g EAA, 1.9 g leucine) to crystalline EAA (7.5 g, 3.0 g leucine) prior to breakfast in older adults, found that EAA supplementation resulted in greater protein and energy intake compared to WP. In addition, consumption of EAAs did not affect appetite ratings or satiety hormones (i.e., PYY), compared with WP [98]. Furthermore, it is pertinent to note that EAA, when ingested with meals, is known to significantly increase the postprandial MPS response by ~30%, but without affecting circulating glucose or insulinemia [99].

In human metabolic research, much like isolated proteins, dosing strategies for EAAs range considerably: from ~1.5 g [100], ~3 g [101,102], ~10 g [103], ~15 g [104] and ~18 g [105] to >20 g [106,107]. Early studies showed that consumption of ~15 g EAAs thrice daily for ~28 days of BR in young males resulted in maintenance of whole-body and leg lean mass compared with the placebo control [36], which was associated with a maintenance of MPS. Importantly, similar observations have also been found following ~10 days of BR in older adults [108]. Thus, supplementing with EAAs in-between meals during periods of BR, as previously established in both younger [36,109] and older adults [108], may offer a viable option for repeated and frequent MPS stimulation, without impacting satiety (Figure 2) [99].

In models of ULI, recent evidence regarding AAs, particularly a novel AA blend with additional non-essential AAs (NEAA)—arginine, glutamine and n-acetylcysteine—has been shown to mitigate muscle atrophy associated with seven days of ULI in healthy young men [110]. Indeed, as the authors stated, it is possible that certain NEAAs may function by attenuating catabolism (i.e., glutamine), maintaining capillary perfusion (i.e., arginine) and reducing oxidative stress (i.e., n-acetylcysteine), which have all been implicated in disuse atrophy [111,112,113,114,115]. Specifically, the consumption of an EAA:NEAA supplement in between meals (3 × 23.7 g = ~71.1 g EAA:NEAA, totalling ~12 g leucine per day) attenuated muscle atrophy and increased intramuscular lipid accumulation compared with a placebo [110]. It is, however, important to note that this study implemented a seven-day loading period of the high-dose EAA:NEAA blend, prior to and during the seven-day period of ULI, which, whilst possible for older adults prior to anticipated disuse events (i.e., elective surgery), would not be possible in acutely hospitalised patients. Furthermore, participants were provided with >70 g AAs on top of a standardised 1.0 g·kg^−1^·day^−1^ of dietary protein. The large amounts of AAs provided by Holloway and colleagues, whilst providing proof of concept, lack real-world practicality [110]. Interestingly, however, leucine-enriched (40%) low EAA dosages of ~3 g (1.2 g leucine, 1.8 g EAA) have been shown to augment similar increases in MPS to that of ~20 g WP (2 g leucine, 7.6 g EAA) in older women [101]. Further, dosages as low as ~1.5 g EAA (0.6 g leucine, 0.9 g EAA) have been reported to stimulate an equivalent MPS stimulation to a large (~40 g) bolus of WP (4 g leucine, 15.2 g EAA) in healthy older women [100]. The addition of ~15 g of EAAs in between, and more importantly, to sub-optimal protein containing meals may therefore be a strategy to mitigate negative effects of disuse and inactivity (Figure 2). This is particularly applicable within in-patient facilities where low-protein consumption and satiety may be of particular concern for older adults.

#### 3.3.2. Leucine

Of the EAAs, the branched-chain AA leucine, has been shown in multiple human models to be a key mediator of postprandial MPS in healthy younger and older adults [90,100,116,117,118], and also in response to acute bouts of RET in the presence of a complete protein source [100,101,119,120]. Leucine has previously been found to have a protective effect on skeletal muscle mass and function during 14 and 7 days of BR in healthy middle-aged [37] and older adults [121], respectively, in the presence of a complete protein source. English et al. (2016) reported that supplementation of a high dose of leucine (0.06 g·kg^−1^**·**BW·meal^−1^ or ~5 g) per meal, attenuated the decline in postabsorptive MPS, muscle mass and muscle strength following 14 days of BR compared with a placebo [37]. Similarly, following seven days of BR in older adults, the same previously mentioned leucine strategy attenuated the loss of leg lean mass; however, no effect was observed for muscle strength or function. Moreover, leucine did not mitigate the effects of BR on muscle fibre CSA or intracellular catabolic protein expression [121]. However, these findings are in agreement with other seven-day ULI protocols which also found no protective effect of leucine supplementation on quadriceps CSA or leg strength [122]. It remains unclear whether the disuse model (BR, ULI or SR), dosage (13.2 g vs. 7.5 g per day) and/or timing of leucine intake (i.e., with or without meals) is attributable to these observed differences and warrants further research.

The role of leucine as a stand-alone nutritional strategy therefore displays equivocal effects at maintaining muscle mass and anabolism. This is particularly important as it is impossible for MPS to exceed MPB when the AA substrates are derived entirely from MPB, and therefore a net positive protein balance cannot occur in the absence of an exogenous supply of a complete EAA profile [123,124]. Nevertheless, ~6 g of leucine containing BCAAs have been previously shown to trigger intracellular anabolic signalling, and augment a small, but significant increase in short-term MPS in younger [125] and older adults [126]. However, in the absence of a full EAA profile, leucine fails to provoke a robust and prolonged state of postprandial MPS as displayed by a traditional ~20 g dose of WP [124]. Therefore, the role of leucine may not be as a stand-alone supplement, but in fortifying complete proteins with additional free-form leucine to augment a greater anabolic effect. Indeed, this has been observed in fortifying low, suboptimal dosages of protein that have been observed to elicit similar acute increases in MPS to that of >25 g of protein [100]. Therefore, it is plausible to suggest that a lower quantity, leucine-enriched protein dose may alleviate some of the satiating effects of a larger protein bolus, as discussed above, whilst still facilitating a net positive protein balance [127], thus supporting the maintenance and/or development of muscle mass in older adults [117,118].

#### 3.3.3. Citrulline

With a lack of evidence to support the use of non-essential AAs (NEAA) in supporting protein accretion, particularly models of disuse [12,13,15,24,128,129,130,131], there is currently only one study to have utilised NEAA, albeit in the form of the single non-essential AA, citrulline [128]. Devries and colleagues postulated that, as a precursor of nitric-oxide, the consumption of citrulline would enhance vasodilatory nutrient delivery to skeletal muscle and attenuate inactivity-induced impairments in MPS in older men [128]. However, purported vasodilatory nutrients have shown little success in augmenting vasodilation, and evidence suggests that even potent pharmacological manipulations of blood flow and tissue-specific microvascular circulation do not enhance postprandial MPS [132,133,134]. Therefore, it is unsurprising that nutrient derived vasodilatory compounds; AAs (i.e., citrulline and arginine) [112,128,135,136,137] and other nutraceuticals with purported vasodilatory properties (i.e., cacao flavanols and Montmorency tart cherry) [138,139] have failed to elicit any notable muscle anabolic effects. Indeed, Devries et al. were unable to demonstrate any effect of daily supplementation of citrulline on femoral blood flow or MPS [128]. Nevertheless, one study has found a beneficial effect of pharmacologically manipulating vasodilation on MPS under conditions of a hyperinsulinemic clamp in older adults [140]. Notably, under normo-insulinemic conditions, such beneficial effects on MPS have not been replicated [141]. The implementation of purported vasodilatory nutrient compounds, therefore, may not be beneficial for enhancement or maintenance of MPS and muscle mass during inactivity and/or disuse in older adults. By direct contrast, vasoconstriction (induced via cold water immersion) has been shown to attenuate the post-exercise increase in MPS by ~20% [142], which may have important implications during disuse and/or periods of hospital stay but warrants further investigation.

### 3.4. Beta-Hydroxy-Beta-Methyl Butyrate (HMB)

Beta-Hydroxy-Beta-Methyl Butyrate (or HMB) is a downstream metabolite of leucine metabolism in skeletal muscle, with a physiological turnover rate of ~0.2–0.4 mg per day or 0.66% of total leucine turnover [143]. HMB, as an oral nutritional supplement, is commercially available in both calcium-salt (Ca-HMB) and free-acid forms [144], both of which have been shown in acute in vitro [145,146,147] and in vivo human models to stimulate MPS and dampen MPB [144,148]. Furthermore, it is also pertinent to note that use of HMB may also be associated with anti-inflammatory effects, although such findings are equivocal [149,150].

Early studies investigating the use of HMB in healthy individuals undertaking RET, demonstrated a highly potent anabolic effect [151,152,153,154]. Specifically, one study observed significant improvements in body composition and muscle strength, which were associated with a ~20% reduction in MPB (assessed via urinary 3-Methylhistidine excretion) and reductions of indirect markers of muscle damage (i.e., creatine kinase and lactate dehydrogenase) [155]. However, more recent RCTs [156,157,158,159] and meta-analyses [160,161] have failed to detect any benefit (i.e., increases in muscle mass, strength or function) of HMB in trained [162] or clinical populations [161]. Nevertheless, HMB is still considered a nutritional compound that may possess the potential to attenuate the rate muscle loss during ageing and disuse [160,163,164,165,166].

The use of HMB supplementation during 10-days BR (1.5 g Ca-HMB, twice daily) has been shown to attenuate muscle anabolic resistance and atrophy compared with a placebo in healthy older adults [165]. Importantly, Ca-HMB in this study, attenuated BR-induced muscle atrophy under conditions of a sub-optimal dietary protein intake [165]. Further, following 10-day BR, the placebo group reported a significant loss of whole-body (~5% vs. 1%), and lower limb (~7% vs. <1%) skeletal muscle mass compared with Ca-HMB, respectively. Similarly, following a standardised liquid meal, whilst postprandial MPS remained unchanged in the Ca-HMB group following 10-day BR, a ~23% reduction in postprandial mixed-MPS was observed in the placebo group [165]. Subsequent follow-up investigations by Standley et al. sought to determine the effects of Ca-HMB on skeletal muscle oxidative function from a sub-sample of older participants, in which mediators of mitochondrial and lipid metabolism were assessed [165,166]. In contrast to previous investigations, Ca-HMB displayed no effect on mitochondrial oxidative phosphorylation, dynamics (*fission and fusion*) or autophagy between Ca-HMB or placebo supplemented groups [166]. However, Ca-HMB supplementation did increase the intramyocellular lipid pool, particularly triacylglycerol lipids, which the authors speculated may have protected skeletal muscle from inflammatory and reactive oxygen species-stimulatory lipids. In vitro data suggests HMB alters myotube lipid biosynthesis and upregulation of fatty acid synthase, which may facilitate anabolic processes and offer protection of muscle cells from other bioactive lipid species [167]. To date, however, the exact mechanisms through which supplemental HMB may act to protect muscle mass during disuse events in older adults remain unclear.

Studies investigating the effect HMB on disuse atrophy, unlike many nutritional strategies, have transitioned into clinically relevant models of critically ill older adults, across a range of patient populations, including patients suffering with malnutrition [168], within intensive care units [169], cachexia patients [170] and patients with HIV [171]. A recent systematic review and meta-analysis conducted by Bear et al. (2019) showed that the data surrounding HMB supplementation are relatively limited (*n* = 13 studies), particularly when provided in isolation and not as part of a mixed nutrient “cocktail” (*n* = 3). For example, HMB is often consumed with other AAs (i.e., arginine and glutamine) or as part of a multi-ingredient oral nutritional supplement (e.g., with additional protein and vitamin D) [161,172,173]. Therefore, as a stand-alone nutritional supplement, more high-quality RCTs are needed to establish HMB’s role in mitigating the loss of muscle mass and strength during disuse events, particularly in older clinical populations.

### 3.5. Creatine Monohydrate

Creatine monohydrate (CM) has been a popular sports supplement with athletic populations for over three decades, with purported significant improvements in muscle mass and strength when combined with RET in young and older adults [174,175,176]. The potential mechanisms through which creatine exerts its beneficial effects are founded on three underlying anabolic constituents. Firstly, CM has been reported to increase osmo-sensing proteins related to signal transduction [177] and targeted mRNA gene expression [178]. Secondly, CM is purported to have an indirect role in muscle anabolism through the phosphorylation of primary downstream anabolic signalling proteins and inhibition of the myokine, myostatin [179]. However, isotopically labelled tracer studies have failed to detect any increases in mixed-muscle, myofibrillar and/or sarcoplasmic MPS following RET and CM supplementation [180,181,182]. However, CM has been reported to have anti-catabolic properties via a reduction in leucine oxidation [181] and urinary 3-methylhistidine excretion [183]. Finally, CM has been suggested to augment muscle satellite cell activation [184,185]. Therefore, with a growing body of literature suggesting that CM can increase muscle mass and strength in older adults [175,176], it has been postulated that CM may also provide some muscle anabolic benefit during periods of musculoskeletal disuse [186,187].

During periods of disuse, Johnston et al. showed that oral CM supplementation (~20 g per day) provided up to three weeks of beneficial effects during a period of upper-arm ULI in healthy younger adults by supporting the maintenance of lean muscle mass, strength, and endurance to a greater extent than a placebo [186]. The potential for CM to attenuate muscle deterioration has also been studied in the context of lower-limb ULI in younger adults [187]. Specifically, following a seven-day loading phase and seven-day maintenance dose pattern of oral CM supplementation (loading Phase: 4 × 5 g day, maintenance: 1 × 5 g day), CM did not attenuate the loss of muscle mass or strength following ~7 days of ULI compared to a placebo control [187]. Whilst, the mechanisms of CM in models of disuse remain unclear, an exploration into the effects of in vivo skeletal muscle creatine sensitivity and transportation may offer insight into the uptake of creatine into muscle cells [188,189]. Nevertheless, the current available evidence regarding its lack of stimulatory effect on MPS, combined with the requirement of a loading phase, suggests that CM supplementation is likely an inadequate and impractical nutritional intervention, at least in isolation, during models of disuse. However, MPS methodologies and disuse interventions have yet to be established in older adults, therefore, we cannot rule out a potential therapeutic benefit of CM in such populations.

### 3.6. Omega 3 (n-3) Fatty Acids

Marine fish oils, particularly n-3 FAs rich in Eicosatetraenoic (EPA) and Docosahexaenoic (DHA), have been shown in both young and older adults to be key signalling mediators of skeletal muscle anabolism [190,191,192], insulin sensitivity [193,194] and inflammation [195]. Human studies utilising intravenous hyperinsulinemic and hyperaminoacidemic clamps have shown a moderate dose (~4 g) of n-3 FA supplementation, augments MPS rates in healthy young [190], middle-aged [190] and older adults [192]. Further, McGlory et al. demonstrated that eight weeks of daily n-3 FA supplementation resulted in a ~23% increase in myofibrillar MPS, compared with a placebo, following a bolus feeding of WP in young men, although this was not statistically significant [196]. However, n-3 FA supplementation was associated with a suppression of resistance exercise and feeding-induced increases in anabolic signalling phosphorylation [196]. In older adults, data suggest ~16 weeks of moderate-to-high dosage of n-3 FA (~4 g·day^−1^) increase the postabsorptive and post-RET (~15–18 h) rates of mitochondrial and myofibrillar protein fractions in healthy older adults [191]. Moreover, long-term supplementation of n-3 FA (~6 months) resulted in significant increase in thigh muscle volume and strength in the absence of RET [197]. Collectively, evidence from these studies forms the basis of n-3 FA supplementation as a potential strategy to counteract disuse atrophy in older adults.

During a 14-day period of ULI, n-3 FA have been shown to attenuate declines in muscle mass in healthy young women compared with an isoenergetic placebo (i.e., sunflower oil) [198]. In addition, daily supplementation of dosages of ~5 g·day^−1^ of n-3 FA resulted enhanced myofibrillar MPS and restored muscle mass over 14 days of reambulation following disuse [198]. The mechanism through which n-3 FA may protect against disuse atrophy might be, in part, explained by an increase in the total phospholipid content of the muscle cell [199]. It is, however, pertinent to note the methodological considerations in the implementation of n-3 FA, in particular, the fundamental loading phase prior to a period disuse. Whilst McGlory and colleagues observed temporal changes in skeletal muscle phospholipid content, a minimum of two weeks (and up to four weeks) of supplementation was required to sufficiently elevate and saturate muscle cell membranes with EPA:DHA [200]. Importantly, it would seem that higher doses of EPA+DHA are required to achieve a more rapid saturation (~2-fold increase in four weeks) [200,201] of muscle cell phospholipids than previous used lower doses (~2-fold increase in eight weeks) [190,192]. However, the temporal changes in skeletal muscle EPA:DHA content have yet to be established in older adults [202]. Moreover, n-3 FA have currently yet to begin translation into older adults and across other experimental models of disuse and inactivity [202].

Interestingly, when considering a food-first approach to nutrient delivery in older adults undergoing disuse events, recent evidence suggests that consuming n-3 FA rich foods offer similar benefits to EPA:DHA status as dietary n-3 FA supplementation [203]. Carboni et al. recently reported thrice-weekly feeding of cooked mussels consumed for lunch over four weeks, resulted in an ~18% increase in n-3 index, as well as a ~40% increase in whole-blood levels of EPA:DHA [203]. Importantly, the EPA:DHA content of cooked mussels in this study was considerably lower (~300 mg) compared with previous investigations (>3000 mg) [190,191,199,200], suggesting that a substantially longer period of time and/or larger doses of n-3 FA rich meals would be required to achieve an optimal n-3 status. Taken together, whilst there is a lack of research across different models of disuse/inactivity, particularly in older patient populations [202,204], the available data suggest a potential benefit of daily n-3 FA supplementation for maintenance of muscle mass in older adults during periods of disuse and/or inactivity, with the caveat that any protective effect may depend on whether a loading phase is possible prior to disuse (i.e., elective surgery patients). However, it is pertinent to note that n-3 FA are also anti-inflammatory and thus, may provide additional benefit to inflammatory disease patients [205,206]. Indeed, lowering inflammation would have a plethora of potential beneficial effects besides maintaining muscle mass [205,206]. However, more studies are required to investigate this further, particularly in models of disuse.

### 3.7. Multi-Ingredient Supplements

In isolation, the use of single-ingredient nutritional compounds, previously mentioned, may not be sufficient to fully attenuate disuse atrophy in both younger and older adults. Nevertheless, a more comprehensive multi-ingredient supplement (MIS) with several purportedly beneficial compounds (i.e., WP, EAAs, creatine, vitamin D, leucine, glutamine, HMB and n-3 FA’s) [207], may be a superior alternative to help support the maintenance of muscle mass and strength during ageing. However, whilst a recent systematic review and meta-analysis encompassing ~35 RCTs utilising MIS as part of a ~6-week training intervention observed MIS as not superior to WP alone in healthy trained populations; MIS was shown to be beneficial in older and untrained adults undertaking a period of multi-modal concurrent exercise [208,209,210]. Furthermore, the majority of studies investigating the use of MIS also implement RET as part of the intervention, and therefore, this may mask any beneficial effects that might been seen with the supplement alone.

Nevertheless, evidence has emerged to demonstrate that a MIS compromising of WP (30 g), creatine (2.5 g), vitamin D (500 IU), calcium (400 mg) and n-3 FAs (1500 mg) may have a beneficial effect on skeletal muscle mass and strength [208,211], inflammation [212] and cognition [213] in older adults, although no effect on exercise-induced myogenesis was observed [214]. MIS, however, did not benefit the adaptive remodelling response to a 12-week multi-modal, concurrent RET and high-intensity interval training intervention in older men [215]. Specifically, Bell and colleagues observed no benefit of twice-daily consumption of a WP-based MIS (WP 30 g, creatine 2.5 g, vitamin D 500 IU, calcium 1500 mg, n-3 polyunsaturated fatty acid 1500 mg) on integrated measures of myofibrillar MPS compared with a carbohydrate-based placebo. This is intriguing as the treatment group consumed ~30–40 g (0.4–0.5 g·kg^−1^·BW·day^−1^) more protein over the experimental period than the carbohydrate control group [208,215]. Although there is currently no evidence supporting the MIS approach both acutely and in older adults during disuse, it is plausible that a blend of multiple beneficial compounds (i.e., EAA, creatine, leucine, HMB, vitamin D and n-3 FAs) may be advantageous in targeting multiple dysfunctional signalling mechanisms associated with disuse and/or inactivity, when combined.

## 4. Conclusions

BR, ULI and SR models can result in dramatic losses of muscle mass, strength and ultimately, functional capacity in a relatively short period of time (2–14 days), with important implications for the functional and metabolic health of older individuals. Although RET interventions are undoubtedly the most potent regulator of muscle protein turnover and promising means of maintaining muscle mass during inactivity, this approach may not always be feasible. Therefore, optimising nutritional intake via high-quality proteins, food-fortification and/or oral nutritional supplements could potentially attenuate disuse-induced impairments in muscle protein turnover that drive the atrophy process. An underlying theme throughout the present review is the lack of translational research from healthy younger and older adults, to frail and hospitalised older patient populations. Current clinical research often includes older adults with high functional capacity and physical activity status, who consume a nutritionally complete diet. Targeting musculoskeletal disuse in older adults with a compromised health status is a stern test of whether or not a nutritional food/ingredient is efficacious, due to the multifaceted nature of mechanical unloading and the progression of sarcopenia. We suggest that a nutrient-rich whole-food dietary approach, providing adequate levels of high-quality protein, is the foremost strategy to protect muscle mass and function in older adults undergoing disuse events. Additional targeted single and/or multi-ingredient supplements may facilitate accrual and retention of muscle tissue during disuse events, and may be a preferable strategy in older adults who are unable to consume adequate high-quality dietary protein from whole-foods alone. Further research should, therefore, seek to determine the temporal change in muscle protein turnover during disuse events and translate promising evidence of potentially beneficial nutritional supplements/ingredients into a clinically relevant setting. This approach will allow researchers to identify optimal times at which to intervene with pragmatic nutritional strategies that can effectively attenuate/prevent muscle metabolic dysfunction, atrophy and impairments in whole-body metabolism in older adults across the health spectrum. Finally, it is worth noting that, in this review, our focus is predominantly on positively influencing MPS and MPB and, thus, not explicitly on the potential beneficial effects that each supplemental strategy might have on other metabolic functions. In this regard, whole-body protein turnover studies need to be conducted to improve our understanding on the impacts to whole-body (protein) metabolism during periods of disuse.
